# Traumatic brain injury causes early aggregation of beta-amyloid peptides and NOTCH3 reduction in vascular smooth muscle cells of leptomeningeal arteries

**DOI:** 10.1007/s00401-025-02848-9

**Published:** 2025-01-22

**Authors:** Ilknur Özen, Sami Abu Hamdeh, Karsten Ruscher, Niklas Marklund

**Affiliations:** 1 Department of Clinical Sciences, Lund Brain Injury Laboratory for Neurosurgical Research, Lund University, 222 20 Lund, Sweden; 2 Department of Medical Sciences, Section of Neurosurgery, Uppsala University, Uppsala, Sweden; 3 Department of Clinical Sciences, Laboratory for Experimental Brain Research, Lund University, Lund, Sweden; 4 Department of Clinical Sciences Lund, Neurosurgery, Lund University, Skåne University Hospital, Lund, Sweden

**Keywords:** Traumatic brain injury (TBI), Vascular smooth muscle cells, Amyloid beta, β-Secretase-derived fragment (βCTF) (C99), NOTCH3

## Abstract

Traumatic brain injury (TBI) often leads to impaired regulation of cerebral blood flow, which may be caused by pathological changes of the vascular smooth muscle cells (VSMCs) in the arterial wall. Moreover, these cerebrovascular changes may contribute to the development of various neurodegenerative disorders such as Alzheimer’s-like pathologies that include amyloid beta aggregation. Despite its importance, the pathophysiological mechanisms responsible for VSMC dysfunction after TBI have rarely been evaluated. Here, we show that acute human TBI resulted in early pathological changes in leptomeningeal arteries, closely associated with a decrease in VSMC markers such as NOTCH3 and alpha smooth muscle actin (α-SMA).These changes coincided with increased aggregation of variable-length amyloid peptides including Aβ_1-40/42,_ Aβ_1-16,_ and β-secretase-derived fragment (βCTF) (C99) caused by altered processing of amyloid precursor protein (APP) in VSMCs. The aggregation of Aβ_1-40/42_ peptides were also observed in the leptomeningeal arteries of young TBI patients. These pathological changes also included higher β-secretase (BACE1) when compared to α-secretase A Disintegrin And Metalloprotease 10 (ADAM10) expression in the leptomeningeal arteries, plausibly caused by hypoxia and oxidative stress as shown using human VSMCs in vitro. Importantly, BACE1 inhibition not only restored NOTCH3 signalling but also normalized ADAM10 levels in vitro. Furthermore, we found reduced ADAM10 activity and decreased NOTCH3, along with increased βCTF (C99) levels in mice subjected to an experimental model of TBI. This study provides evidence of early post-injury changes in VSMCs of leptomeningeal arteries that can contribute to vascular dysfunction and exacerbate secondary injury mechanisms following TBI.

**Supplementary Information:**

The online version contains supplementary material available at 10.1007/s00401-025-02848-9.

## Introduction

Traumatic brain injury (TBI) elicits a range of secondary injury mechanisms that markedly exacerbate the injury, both at short and long term. Since the energy metabolic demands of the brain increase early post-injury, cerebrovascular abnormalities profoundly influence the development of the initial injury. Secondary injury events such as ischemia and hypoxia are common in TBI and can negatively affect patient outcomes [14]. Thus, the maintenance of cerebral blood flow (CBF) autoregulation and ensuring sufficient oxygen delivery is a crucial factor in the management of patients with TBI, particularly in the early phases of the injury [60, 61]. TBI can trigger a series of cerebrovascular events including loss of vascular tone [11, 63] and a reduced CBF [31, 57]. In addition, arteriolar pathology is involved in vascular dysfunction, potentially leading to white matter hyperintensities, hypoxia and ischemia, as well as microinfarcts [5, 24]. Finally, TBI is a risk factor for the development of neurodegeneration [22], to which pathological cerebrovascular alterations could be a main contributor [1].

Vascular smooth muscle cells (VSMCs) are the major contractile components of the cortical and leptomeningeal arteries in the brain and play a crucial role in regulating CBF under normal conditions. In moderate to severe human TBI, vascular tone can be altered causing reductions in CBF [11, 64]. Therefore, it is essential to understand the potential changes to the VSMCs in TBI to minimize secondary injury to the brain. However, the cellular and molecular mechanisms that contribute to VSMC pathology in leptomeningeal arteries after TBI have not been previously evaluated.

In the present study, we focused on the impact of TBI on the changes of VSMCs of leptomeningeal arteries. We show that TBI resulted in a significant decrease in VSMCs markers including NOTCH3 and alpha smooth muscle actin (α-SMA). This was closely associated with increased aggregation of variable-length amyloid beta peptides in the leptomeningeal arteries, observed also in young TBI patients. The increase in amyloid beta peptides in VSMCs resulted from a TBI-induced imbalance of α-secretase A Disintegrin And Metalloprotease 10 (ADAM10) and β-secretase (BACE1) levels, potentially caused by hypoxia and oxidative stress as evident from our in vitro studies. We further validated decreased expression of NOTCH3 using an in vitro model of human VSMCs and in a clinically relevant mouse model of diffuse TBI.

## Materials and methods

### Ethical statement for human samples

All clinical and experimental research described herein were approved by the regional ethical review board (decision numbers 2005/103, 2008/303, 2009/89, 2010/379 and 2020-03154). Written permission was obtained from the TBI patients’ relatives in the acute phase and from the patients themselves if they recovered sufficiently from their injury at ca 6 months post-injury. Similarly, written consent was obtained from each control patient prior to inclusion in this biobank.

All patients had severe TBI defined as a post-resuscitation Glasgow Coma Scale (GCS) score ≤ 8. The TBI patients were subjected to surgical focal decompression and removal of contused TBI tissue due to life-threatening elevations of intracranial pressure and/or the presence of a space-occupying brain swelling or haemorrhage. All patients received neurocritical and neurosurgical care, followed by neurorehabilitation. The total number of patients in this cohort was *n* = 24. However, *n* = 9 patients were initially excluded from the analyses due to a lack of leptomeningeal arteries. The characteristics of the included TBI patients (*n* = 15; age range 19–74 years) and controls included in the study are shown in Table S1. At the time of follow-up, one patient was deceased.

Control human subjects (*n* = 5) were obtained from the Uppsala Biobank/archived at the Department of Clinical Pathology and Cytology. The five frontal samples were derived from uninjured patients, ranging from 43 to 63 years of age, without previous history of TBI or neurodegenerative disorders and who had died from systemic, unrelated causes. The postmortem time of the included control patients (time from death to tissue fixation) was 40 + / − 17 h (range 10–48 h).

### Human sample preparation.

For immunohistochemistry (IHC) analyses, surgically resected brain tissue samples were placed in 4% buffered formalin (Histo-Lab Products AB, Gothenburg, Sweden, catalog no: 02176). Following 24–72 h fixation time, the samples were paraffin-embedded and processed by hardware Tissue tek VIP (Sakura, CA, USA, RRID:SCR_020858). Seven μm thick sections were cut and placed on SuperFrost^®^ plus slides (Menzel-Gläser, Vienna, Austria) for immunohistochemical analysis.

### Deparaffinization, dehydration, and antigen retrieval of human sections

The sections were deparaffinised in 2 × changes of xylene and rehydrated in 2 changes in 100% alcohol for 3 min each, once in 95% alcohol for 3 min, once in 80% alcohol for 1 min. Afterwards, they were washed in deionized water and placed in antigen retrieval solution. Heat-mediated antigen retrieval was performed in 10 mM citrate buffer (pH 6.0) for 40 min at 80 °C before primary antibody incubation.

### 3,3′-Diaminobenzidine (DAB) staining for human brain samples

Human brain sections were quenched with 3% H_2_O_2_ peroxidase solution for 15 min, then blocked in phosphate buffer saline (PBS) supplemented with 0.5% Triton X-100 and 3% normal Donkey Serum Cat. No:017-000-121, Jackson Immunoresearch, Baltimore, USA, RRID: AB_2337258) at room temperature (RT) for 1 h. Afterwards, the sections were incubated in primary antibodies diluted in blocking solution at 4 °C overnight. Primary antibodies used: mouse MOAB-2 (pan Aβ) (1:2000, Merck-Millipore, Cat. No: MABN254, RRID: AB_2895168), mouse Anti-β Amyloid 1–16 (6E10) (1:1000, BioLegend, Cat. No:803015, RRID: AB_2728527). Following washing steps in 0.5% Triton-X100 in PBS (PBS-TX) three times, sections were incubated with corresponding anti-rabbit biotinylated secondary antibody (1:500, 711-065-152, Jackson Immunoresearch, Baltimore, USA, RRID: AB_2340593), in 3% normal donkey serum (NDS) PBS-T, at RT for 2 h, and the signal was enhanced by using Vectorstain ABC Elite kit (Cat. No: PK6100 Vector Laboratories, CA, USA, RRID: SCR_000821). Staining was revealed using chromogen 3,3-diaminobenzidine-tetrahydrochloride and 3% H_2_O_2_ using DAB Substrate Kit (Cat. No: VECTSK4100, Vector Laboratories, CA, USA, RRID: SCR_000821). Sections were dehydrated in consecutive higher concentrations of ethanol, followed by xylene and mounted using Pertex (Histolab AB, Gothenburg, Sweden). The stained sections were analysed using Olympus BX51 light microscope (RRID: SCR_018949) and CellSens digital imaging software (RRID: SCR_014551). The figures were composed using Adobe Photoshop 25.1.0. software (RRID: SCR_014199).

### Immunofluorescence staining

Immunocytochemistry (ICC): human brain vascular smooth muscle cells (HBVSMCs) were plated on glass coverslips coated with Poly-L-lysine (P4832, Sigma-Aldrich, RRID: SCR_008988, 90 µg/ml).The cells were fixed with 2% paraformaldehyde (PFA, Sigma-Aldrich, RRID:SCR_008988) 10 min at RT, washed with PBS three times. Next, the cells were incubated 5% donkey serum in PBS for 1 h at RT. The cells were incubated with primary antibodies at 4 °C overnight and secondary antibodies at 4 °C for 2 h in the presence of 0.1% Triton X-100 (Sigma-Aldrich, RRID:SCR_008988) and 2% donkey serum.

*For Immunohistochemistry (IHC):* human brain Sects. (7 μm) and coronal mouse brain Sects. (30 μm) were blocked for 1 h in PBS-TX supplemented with 3% NDS (G9023, Sigma-Aldrich, RRID: SCR_008988) after being washed in PBS three times. The sections were incubated with primary antibodies diluted in blocking solution overnight at 4 °C. The sections were mounted on slides after three final washes in PBS. Primary antibodies used for ICC and IHC: rabbit BACE1 (1: 500, ThermoFisher, Cat. No:PA5-19,952, RRID: AB_11153759, goat BACE1 (1: 200, ThermoFisher, Cat. No: PA1-20,215, RRID: AB_557693), rabbit ADAM10 (1: 200, ThermoFisher, Cat. No: PA6-119,642, RRID: AB_2913215), rabbit NOTCH3 /N3ICD (1: 200, Abcam, Cat. No: ab23426, RRID: AB_776841), mouse NOTCH3/N3ECD (1:200, Merck-Millipore, Cat. No: MABC594, RRID: AB_2890101), goat podocalyxin (1: 400, R&D Systems, Cat. No: AF1658, RRID: AB_354920), mouse smooth muscle actin (Clone, 1A4) (1:500, DAKO, Cat. No: M0851, RRID: AB _2223500), mouse APP-C99 Antibody, clone mC99(70–80) (1: 100, Merck-Millipore, Cat. No: MABN380, RRID: AB_2714163), Phalloidin-iFluor 488 (1:1000, Abcam, ab176753, RRID: SCR_012931), mouse anti-PCNA (1:1000, Novus Biologicals, USA, RRID: SCR_004286), prolyl 4-hydroxylase-2 (PHD2) (1: 1000, ThermoFisher, Cat. No: PA5-78,511, RRID: AB_3674163). The secondary staining was conducted using species-specific fluorophore-conjugated antibodies (Streptavidin Alexa 488, Molecular Probes; Cy3 or Cy5, Jackson Immunoresearch Labs, RRID: SCR_010488).

### Confocal microscopy image and analyses

The leptomeningeal arteries with a diameter larger than 20 μm were imaged at × 40 objective, using Zeiss LSM 780 confocal laser scanning microscope, RRID: SCR_020922. The figures were composed using Adobe Photoshop 2024 software(RRID: SCR_014199). Image J version Fiji Mac version Java 1.8.0_345 software NIH, Bethesda, USA, RRID: SCR_003070) was used for the quantitative analysis of the leptomeningeal arteries to measure the percentage (%) and raw intensity of NOTCH3, α-SMA, ADAM10, and BACE1 within the total area of leptomeningeal artery wall. The perimeter of each selected arteries was traced by hand and the area of whole vessel wall was calculated using the Image J region of interest manager and a make band section. The co-localization of each fluorescence colour was calculated using colour thresholds selected in pairs to calculate the number of pixels overlapping two colour channels. The Image J analysis tool was used to quantify the area covered by each fluorescence colour and each pair of colours. The individual fluorescence values were calculated as a percentage of the total vessel wall area.

### Human brain vascular smooth muscle cells (HBVSMCs)

HBVSMCs were purchased from ScienCell Research (Cat. No: 1100, Carlsbad, California, USA, RRID: SCR_026150). The smooth muscle cell medium was composed of 2% foetal bovine serum, 1% smooth muscle cell growth supplement and 1% penicillin/streptomycin solution (all purchased from ScienCell Research Laboratories, Carlsbad, California, USA, RRID: SCR_026150).

### Oxygen–glucose deprivation/reoxygenation (OGD/R)

HBVSMCs were seeded in 6-well plates exposed to oxygen deprivation conditions generated in a humidified, gas-tight hypoxia chamber incubator (Electrotek Ltd, Keighley, UK, RRID: SCR_026207) with a gas composition of 85% N_2_, 5% CO_2_ and 10% H_2_. The OGD medium was equilibrated to the ambient gas composition in the hypoxic chamber for 30 min resulting in oxygen levels of 0.5–0.7% when added to the cells. During experiments, an indicator solution was placed in the chamber. After OGD, cells were re-oxygenated for 23 h. Prior to re-oxygenation, the cells were washed in PBS and OGD medium was replaced with the smooth muscle cell medium containing 2% foetal bovine serum.

For BACE1 inhibition experiments, the cells were treated with 1 µM BACE1 inhibitor LY2886721 (Selleckchem S2156, Boston, MA, RRID: SCR_003823) during OGD/R exposure [45].

### Central fluid percussion model (cFPI)

Adult male mice C57BL/6 mice (pre-injury minimum weight 22 gr, 8–11 weeks old, Taconic, Denmark), were housed with free access to food and water. All procedures on research animals were in accordance with the European Union directive (2010/63/EU) and approved by the local ethical committee at Lund University as well as the Swedish Department for Agriculture (Jordbruksverket; Dnr: 5.8.18-13,263/2022).

The surgical procedure for central fluid percussion injury (cFPI) has been performed to obtain frozen mouse tissue samples for Western blot analyses as previously described [50]. In brief, mice were randomly subjected to sham injury (*n* = 8) or cFPI, which has been described in detail previously (cFPI; *n* = 10) [52]. In addition, five naïve, non-injured mice were included. The mice were anaesthetized in a ventilated plexiglass chamber with 4% isoflurane and 3.0 mm diameter craniotomy was made over the midline by keeping the dura mater and the superior sagittal sinus intact. Then, a plastic cap was placed on craniotomy filled with isotonic saline at room temperature and attached to the Luer-Lock on the fluid percussion device (VCU Biomedical Engineering Facility, Richmond, VA). The fluid percussion pendulum was released to create a pressure wave subsequently transmitted into the cranial cavity. There were immediate post-injury seizures in all animals post-cFPI, and the post-injury apnoea, observed in all cFPI-injured mice, was 53 ± 15 s (range 35–80 s). One mouse died due to accidental injury to the superior sagittal sinus from drilling. Two cFPI animals died at time of impact resulting in injury-related mortality, however there was no delayed post-injury mortality. Sham-injured animals were subjected to anaesthesia and surgery including craniotomy, but the pendulum was not released. After surgery, the cap was removed, the bone flap replaced, and the skin sutured using resorbable sutures.

To obtain representative pictures of NOTCH3 in mice, we used cortical sections (*n* = 2 for Sham, *n* = 2 cFPI) from our previous study, which focused on different objectives and brain regions (the cerebellum) [52]. This is in line with the guideline principles at Karolinska Institute, with the aim to reduce the number of animals used in testing according to the 3R principle.

### Mouse tissue preparation for IHC

Mouse brain tissue was prepared as described previously [52]. Briefly, the mice were sacrificed at 2 dpi and transcardially perfused using 4% formaldehyde (Merck, Darmstadt, Germany, RRID: SCR_001287). The brains were post-fixed overnight at 4 °C and placed in 25% sucrose solution. The brains were sectioned at 30 μm thickness and stored in anti-freezing buffer at − 20 °C.

### Western blotting

The upper surface of the cortical area including leptomeningeal and penetrating arteries were micro-dissected and were homogenized by sonication in lysis buffer (20 mM Tris pH 7.5, 150 mM NaCl, 1 mM EDTA, 1 mM EGTA, 1% Triton X-100, 1 mM β-glycerolphosphate, 1 mM sodium orthovanadate (Na_3_VO_4_), 1 mM phenylmethylsulfonyl fluoride (PMSF) and Complete™ Protease Inhibitor Cocktail (4,693,116,001, Sigma-Aldrich, RRID: SCR_008988) and centrifuged at 14.000 RPM for 20 min at 4 °C. The supernatant was collected and kept at − 80 °C. The protein samples were boiled for 5 min in 2 × Laemelli buffer supplemented with 10% 2-mercaptethanol. Protein (20 μg) separation was performed on Mini-Protean^®^ TGX^™^ precast gels (Bio-Rad, Hercules, USA, RRID:SCR_008426); afterwards proteins were transferred onto PVDF membranes using a Trans-blot^®^ Turbo™ (Bio-Rad, Hercules, USA, RRID:SCR_008426) system. Membranes were blocked in TBS (20 mM Tris, 136 mM NaCl, pH 7.6) supplemented with 0.1% Tween 20 and 5% non-fat dry milk before incubation with primary antibodies overnight at 4 °C. Signals were boosted by binding of horseradish peroxidase (HRP)-linked secondary antibodies (anti rabbit 1:25,000 and anti-mouse 1:10,000, Sigma-Aldrich, Cat No: A0545, RRID: AB_257896) to the primary antibodies. The membranes were stripped and re-probed for β-actin (1:75,000, Sigma-Aldrich, Cat No: A3854, RRID: AB_262011)) or GAPDH (1:50,000, Sigma-Aldrich, Cat No: G9295, RRID: AB_1078992). Membranes were exposed on a ChemiDoc™ MP system (BioRad, RRID: SCR_021693) using a chemiluminescence kit (11,363,514,910, Merck Millipore, Billerica, MA, USA, RRID: SCR_001287) that reacted with the HRP-linked secondary antibodies. The densitometry analyses were conducted using ImageJ software (MA, USA, RRID: SCR_003070) and protein levels were calculated as percentage of β-actin or GAPDH expression.

### Statistics

Graphs and statistical analysis were made with GraphPad Prism 10 (GraphPad Software, La Jolla, CA, USA, RRID: SCR_002798). The experimental sample sizes were selected via power analysis of preliminary data using an online calculator: https://www.gigacalculator.com/calculators/power-sample-size-calculator.php. The Shapiro–Wilk normality test was used to evaluate normality of data distribution. For statistical analyses of Western blots related to experimental TBI and in vitro BACE1 experiments, Kruskal–Wallis one-way ANOVA with Dunn’s post-test for non-normally distributed data or one-way ordinary ANOVA with Tukey’s post-test for normally distributed data were used. Statistical analyses for NOTCH3, α-SMA, ADAM10, and BACE1 expression in the leptomeningeal arteries were performed with Student’s t test between human TBI and control subjects. All data are expressed as the mean ± S.E.M. Significance was set at *P* < 0.05.

## Results

### Human TBI causes abnormalities in NOTCH3 expression in the vascular smooth muscle cells of leptomeningeal arteries

**Fig. 1:**
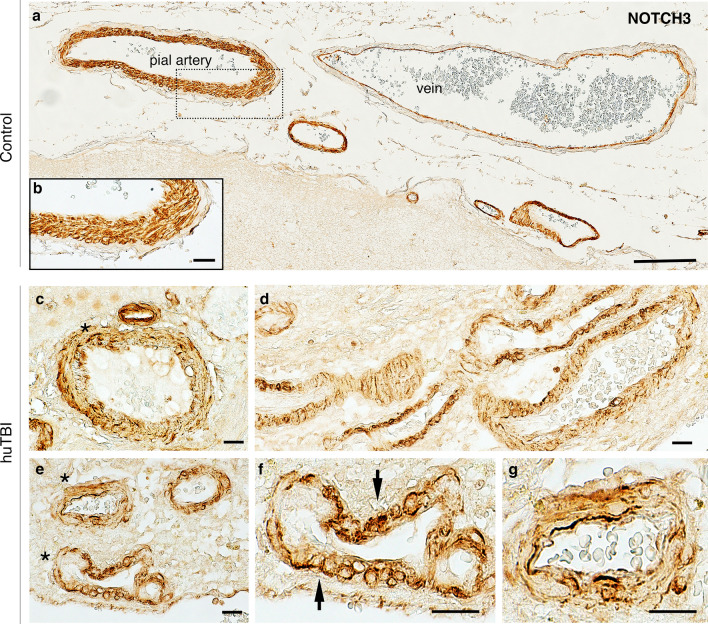
Altered NOTCH3 expression in leptomeningeal arteries of human acute TBI subjects. NOTCH3 expression in the leptomeningeal arteries of **a**, **b** control subjects and **c**–**g** brain samples surgically resected from severe, acute human TBI patients. **a** Representative bright-field images of NOTCH3 in leptomeningeal arteries of different sizes in control subjects. **b** The boxed area in **a** showing NOTCH3 staining through the full thickness of tunica media of artery walls of the control subject. **c–g** Transverse and longitudinal views of leptomeningeal arteries of acute human TBI patients showing a partial circumference abnormality of NOTCH3 expression in the tunica media (asterisks in **c**, **e**, and arrows in** f**). Scale bars = 200 μm in (**a**), 20 μm in (**b**–**g**)

**Fig. 2:**
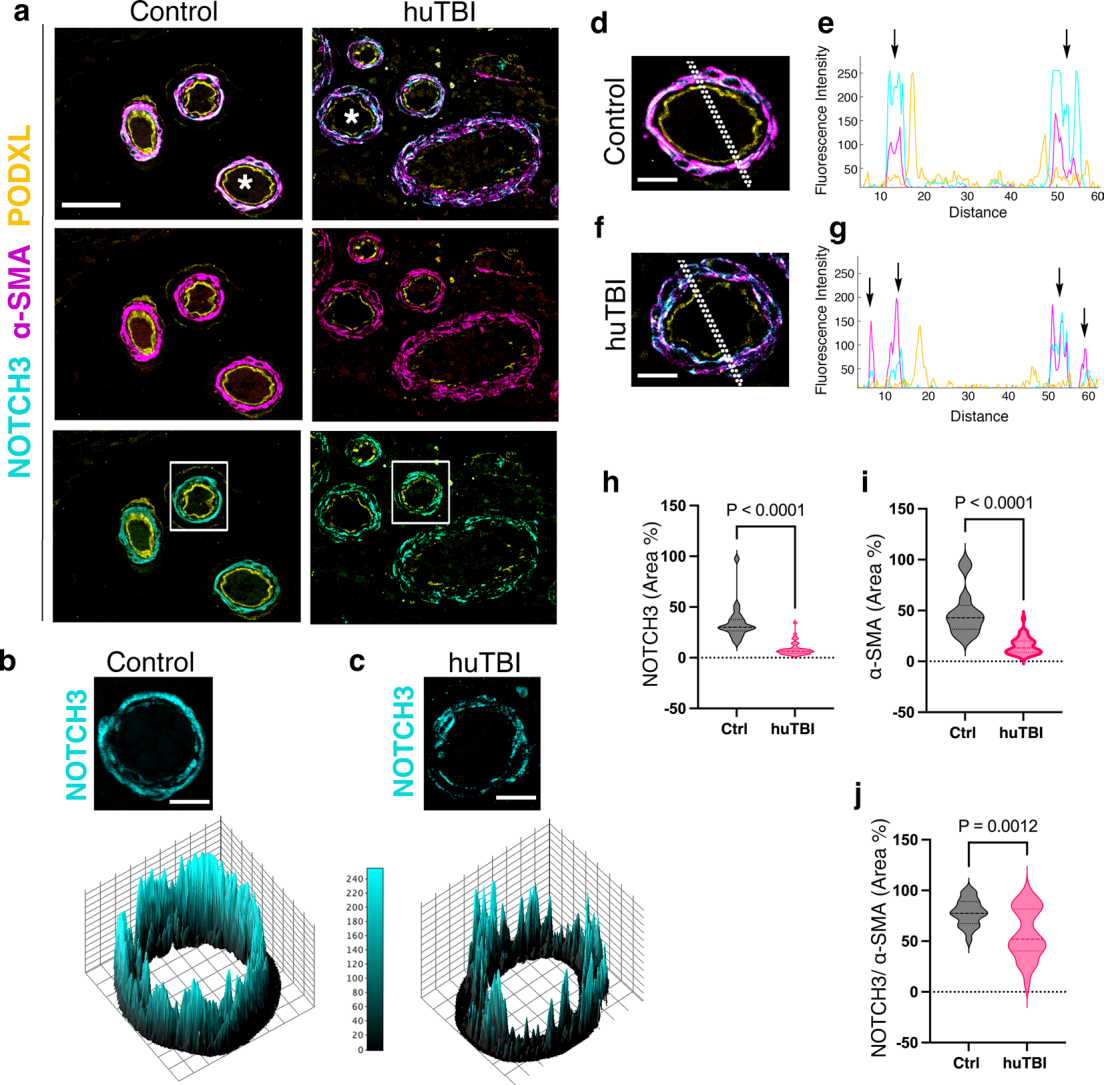
Acute human TBI-induced NOTCH3 changes are specific to vascular smooth muscle cells in the tunica media. **a** Representative confocal images of triple staining of NOTCH3 (cyan), α-SMA (magenta), and podocalyxin (yellow) in the leptomeningeal arteries of control and human TBI subjects. **b**, **c** Interactive 3D surface plots of individual leptomeningeal arteries in boxed areas in **a** showing spatial intensity profile of NOTCH3 expression in the tunica media. **d**–**g** Higher magnification confocal images of leptomeningeal arteries (white asterisks in a) and the fluorescence intensity profiles of NOTCH3, α-SMA, and podocalyxin expression. **h**, **i** Quantification of NOTCH3 (**h**), α-SMA (**i**), NOTCH3/ α-SMA co-labelling (**j**) expressed as a percentage (%) of tunica media of leptomeningeal arteries of control and human TBI subjects (*n* = 22 arteries, control subjects, *n* = 46 arteries in TBI patients, Mann–Whitney test, Mean ± SEM). Scale bars = 50 μm in (**a**), 10 μm in (**b**, **c**), 20 μm in (**d**, **f**)

NOTCH3 is highly expressed in VSMCs in arteries and is involved in modulation of the contractile phenotype of VSMCs as well as their vascular tone [8, 15]. Therefore, we first investigated how human TBI influences NOTCH3 levels in the tunica media of leptomeningeal arteries. In the human control subjects, strong reactivity to NOTCH3 was observed throughout the entire thickness of the tunica media, consisting of multiple layers of VSMCs and around the whole circumference of the walls of arteries with different sizes in the subarachnoid space (Fig. 1a, b). However, the expression pattern of NOTCH3 in leptomeningeal arteries was severely disrupted in the human TBI samples. (Fig. 1c–g). Contrary to VSMCs of tunica media in control subjects, the sections from TBI patients showed an irregular and disintegrated NOTCH3 staining pattern with an accumulation of vacuolar-like structures (Fig. 1c-g). DAPI staining was performed for nuclear visualization (Fig. S1). To examine alterations in expression pattern of NOTCH3 in the VSMCs of leptomeningeal arteries, triple co-staining was performed using alpha smooth muscle actin (α-SMA), a marker for VSMCs, along with podocalyxin for endothelial cells in the intima (Fig. 2a). In control subjects, NOTCH3 and α-SMA expression were evenly distributed in the soma of vascular smooth muscle cells in the tunica media of leptomeningeal arteries (Fig. 2b, d, e). In TBI patients, NOTCH3 expression in the tunica media of leptomeningeal arteries was mainly located on the surface of vascular smooth muscle cells that showed a ballooned-like morphology where NOTCH3 and α-SMA expression showed two peaks, lower than in control subjects (Fig. 2c, f, g). There was a fivefold decrease in NOTCH3 (*p* < 0.0001) (Fig. 2h) and a 3.2-fold decrease in α-SMA expression (*p* < 0.0001) (Fig. 2i) in the tunica media of leptomeningeal arteries of human TBI patients compared with control objects. Similarly, there was a significant (*p* = 0.0012) decrease in the percentage area of NOTCH3 co-localizing with α-SMA in tunica media of leptomeningeal arteries in TBI when compared to control subjects (Fig. 2j).

### Presence of amyloid beta peptides in degenerating vascular smooth muscle cells with decreased NOTCH3 expression in human TBI samples

**Fig. 3:**
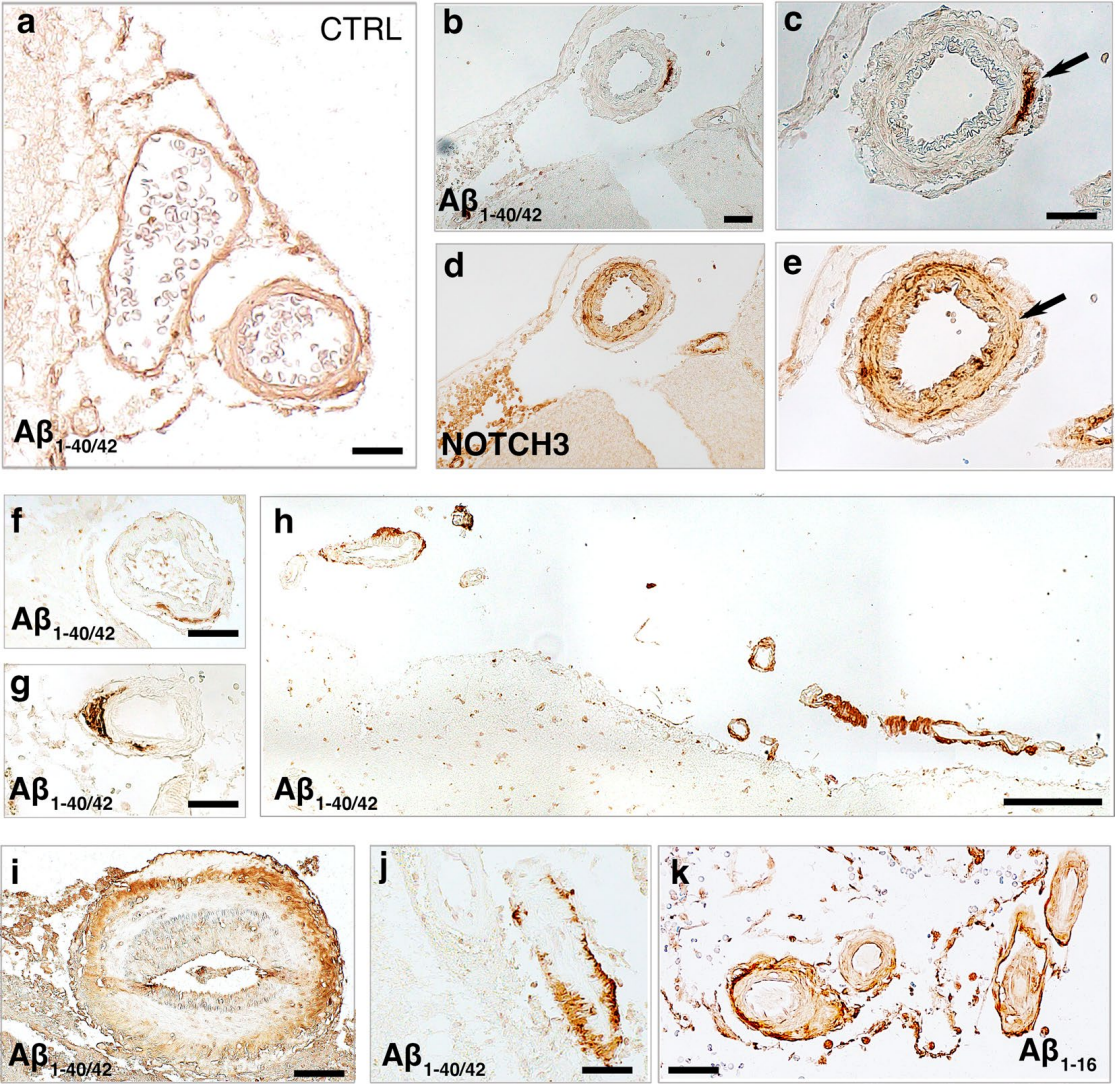
Amyloid beta peptides in the leptomeningeal arteries of human acute TBI subjects. **a**–**c** Representative bright-field images of Aβ_1-40/42_ (MOAB-2) of leptomeningeal arteries in human acute TBI sections compared with control subject. Aβ_1-40/42_ deposits in the tunica media where NOTCH3 expression decreased (**b**–**c**, arrows). Transverse and longitudinal views of leptomeningeal arteries with different sizes showing patchy Aβ_1-40/42_ (MOAB-2) (**a**–**c**, **f**, **g**, **i**–**k**) and Aβ_1-16_ (6E10) (**k**) peptides around tunica media (**a**–**c**, **f**, **g** and **i**) or as a thin rim spanning the entire vessel wall circumference (**h**, **j**, and **k**). Scale bars = 50 μm in (**a**–**g**, **i**–**k**), 200 μm in (**h**)

We next analysed whether Aβ peptides are found in leptomeningeal arteries in acute human TBI (Fig. 3a–f). Amyloid beta peptides were detected by performing DAB staining with a pan antibody to Aβ_1−40/42_ (MOAB-2, clone 6C3) that recognize oligomeric and fibrillar forms of Aβ_42_ and Aβ_40._ The accumulation of Aβ was predominantly found in the tunica media and around leptomeningeal arteries of TBI subjects compared with control subjects (Fig. 3a–g, i). However, Aβ peptides in the outermost part of the adventitia of leptomeningeal arteries were observed in only three out of the 15 subjects, corresponding to 20% of the cohort (Fig. 3h, j, k). These findings were further supported by immunostaining with an antibody specific to N-terminally truncated Aβ_1-16_, clone 6E10 (Fig. 3k).

**Fig. 4:**
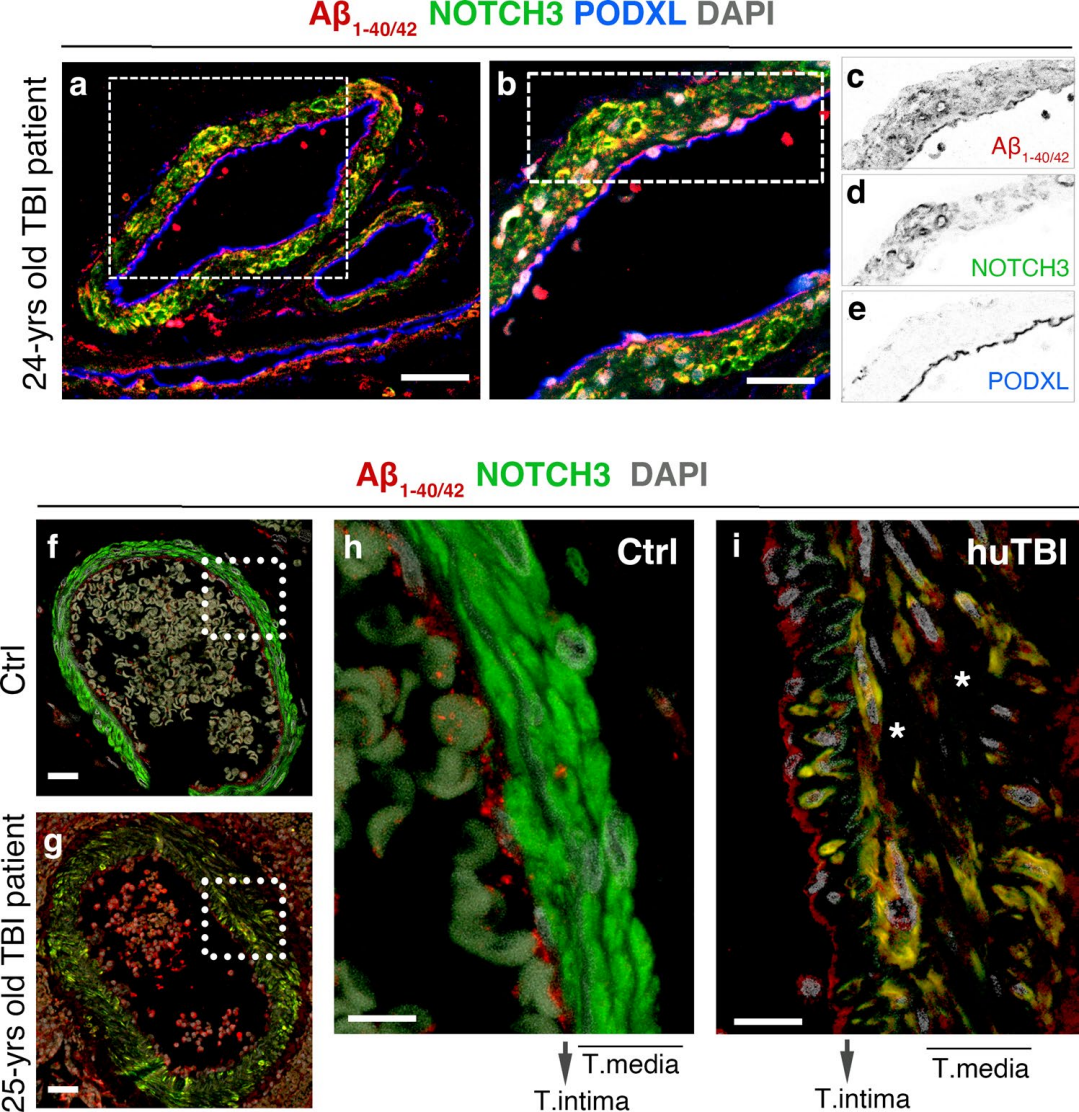
Accumulation of beta-amyloid peptides is associated with changes in NOTCH3 expression in leptomeningeal arteries. **a** Confocal images of triple staining of Aβ_1-40/42_ (red), NOTCH3 (green), and podocalyxin (blue) in the leptomeningeal arteries of a 24-years old TBI patient. Scale bars = 50 μm (**b**) Higher magnification of boxed area in (**a**) showing changes in the expression of Aβ_1-40/42_ (red), NOTCH3 (green), and podocalyxin (blue) in vascular smooth muscle cells of tunica media. **c**–**e** Confocal pictures in the boxed area (**b**) are inverted into greyscale. **f**, **g** Representative confocal images of double staining of NOTCH3 (green), Aβ_1−40/42_ (red) in medium-sized and large leptomeningeal arteries of control and human TBI subjects. Scale bars = 20 μm. **h**–**i** Higher magnification confocal images of large leptomeningeal arteries in a 25-years old TBI patient (white boxed in **f** and **g**) showing a decrease in NOTCH3 (green) signalling in the vascular smooth muscle cells (VSMCs) and Aβ_1-40/42_ accumulation in the VSMCs. Scale bars = 10 μm in (**h**, **i**)

We then performed confocal microscopy to investigate the interrelationships between deposition of Aβ and changes in NOTCH3 expression in the leptomeningeal arteries. We found that decreased NOTCH3 levels coincided with Aβ peptides in leptomeningeal arteries, even in the brain tissue of a 24-year-old TBI subject, the youngest included patient (Fig. 4a–e). The aggregation of Aβ peptides was primarily found on the vascular smooth muscle cells with decreased NOTCH3 levels in the tunica media of TBI samples (Fig. 4a, g, i). The confocal imaging analyses on another young patient, a 25-year-old TBI subject, showed a loss of vascular smooth muscle cells in the tunica media and a lack of NOTCH3 expression and localized Aβ_1-40/42_ peptides in the TBI samples compared to control (Fig. 4f, i).

### Human traumatic brain injury leads to changes in expression of ADAM10 and BACE1 in leptomeningeal arteries

**Fig. 5:**
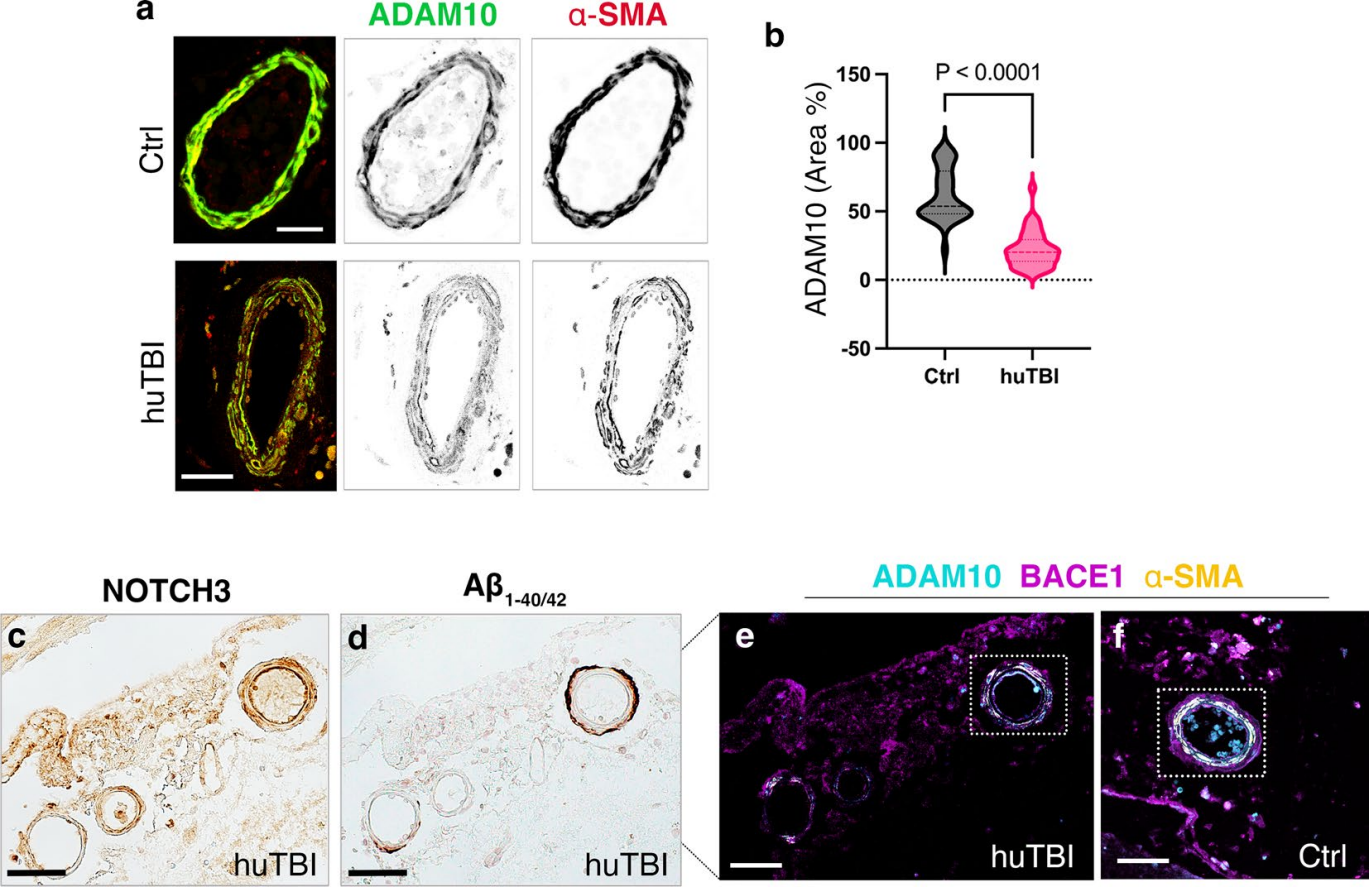
ADAM10 levels decreases in the leptomeningeal arteries following acute human TBI. **a** Confocal images of staining of ADAM10 (cyan) and α-SMA (yellow), which were inverted into greyscale in boxes, in the leptomeningeal arteries of control and human TBI subjects. Scale bars = 20 μm. **b**–**c** Quantification of ADAM10/ α-SMA co-labelling (**c**) expressed as a percentage (%) of tunica media of leptomeningeal arteries of control and human TBI subjects (n = 38 arteries, control subjects, n = 41 arteries, Mann–Whitney test, Mean ± SEM) (**f**) A representative bright-field image showing dense Aβ deposition covering the whole wall of the medium-sized leptomeningeal arteries in the human acute TBI subject. **g**, **h** Immunofluorescence staining of ADAM10 (cyan), BACE1 (magenta), and α-SMA (yellow) in the medium-sized leptomeningeal artery (boxed in f) of (**g**) human TBI and (**h**) of control subjects. Scale bars = 50 μm

Amyloid beta is generated by sequential proteolytic cleavage of the APP through two separate pathways: i) the non-amyloidogenic pathway is initiated by the α-secretase enzyme disintegrin–and-metalloproteinase domain-containing protein 10 (ADAM10), prior to cleavage by γ-secretase; ii) in the amyloidogenic pathway, APP is cleaved by a protein-cleaving enzyme 1 (BACE1), the main β-secretase enzyme, and γ-secretase. ADAM10 is one of the major metalloproteinases that regulate the expression and the proteolytic processing of NOTCH3 [25]. Therefore, we specifically focused on ADAM10 in the tunica media labelled with α-SMA (Fig. 5a). There was a 60% (*p* = 0.0012) decrease in the percentage area of ADAM10 co-localized with α-SMA in tunica media of leptomeningeal arteries from TBI compared with controls, regardless of the presence of Aβ peptides and the size of the leptomeningeal arteries (Fig. 5b). When we examined the arteries containing high Aβ peptides on their outer wall, which is 20% of the cohort (Fig. 5c, d); we observed a decrease in NOTCH3 and α-SMA in VSMCs in leptomeningeal arteries (Fig. 5e, f). Overall, human TBI resulted in a 76% decrease in the expression of ADAM10 (*p* < 0.0001), while there was only a 40% decrease in BACE1 levels (*p* < 0.0001) in the tunica media of VSMCs compared with controls.

### Hypoxia/oxidative stress results in decreased expression of NOTCH3, altered amyloid beta processing and imbalance of ADAM10/ BACE1 levels in the VSMCs after TBI

**Fig. 6:**
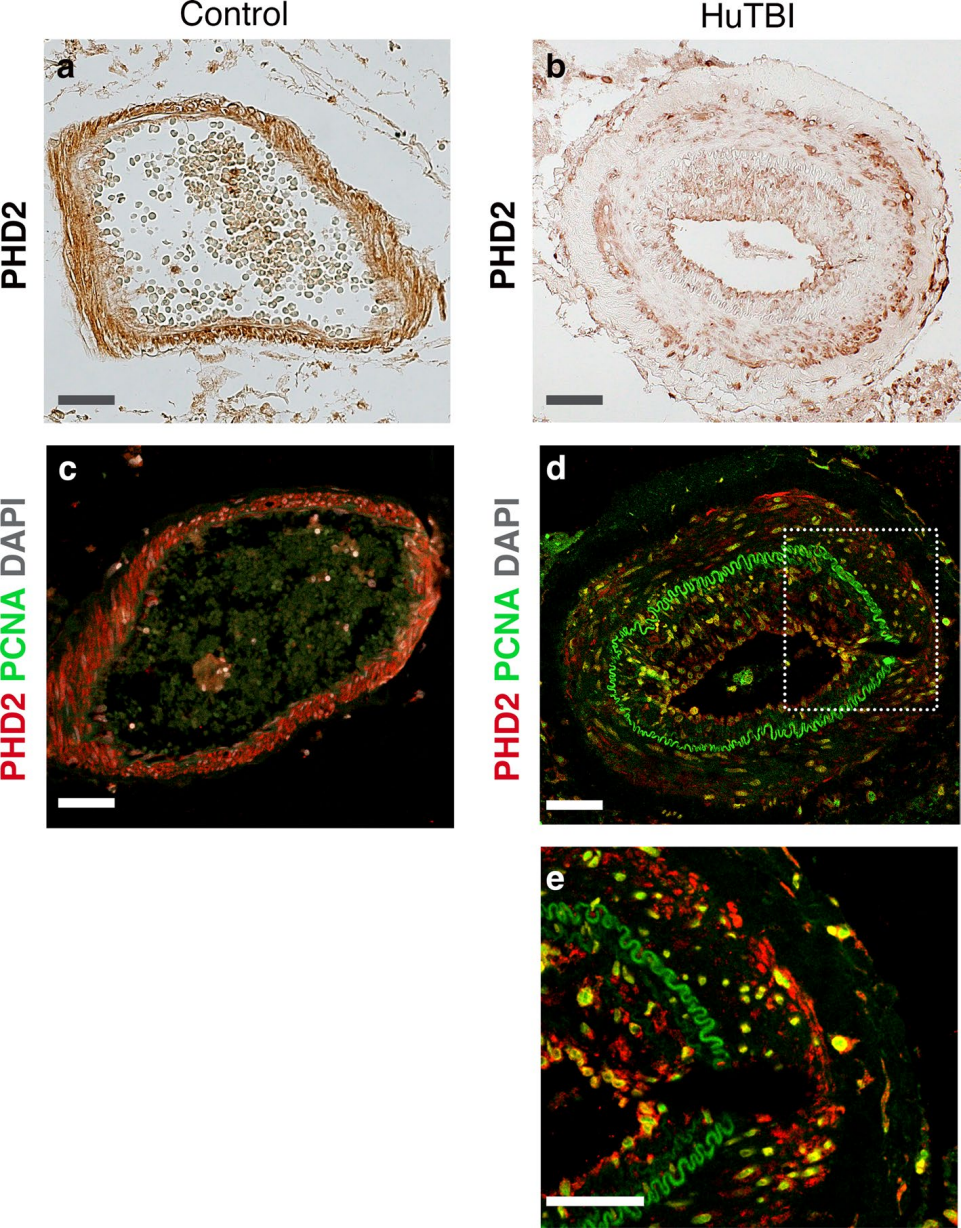
Human TBI increases hypoxia and proliferation in the VSMCS of leptomeningeal arteries. **a**, **b** Bright-field images of prolyl 4-hydroxylase-2 (PHD2) of leptomeningeal arteries in human acute TBI sections compared with control subject, scale 20 μm. **c**, **d** Confocal images of double staining of PHD2 (red) and PCNA (green) in leptomeningeal arteries of control and human TBI subjects, scale 20 μm. DAPI (grey) was used as counterstaining. **e** Higher magnification confocal image of the boxed area in large leptomeningeal arteries from human TBI subject showing co-expression of PCNA and PHD2, scale 50 μm

Early secondary brain injury factors including hypoxia and hypotension are common in the initial post-injury hours following severe TBI [32]. In TBI, oxygen-sensing systems are crucial for adjusting CBF by facilitating arterial vasodilation and constriction [66], which are primarily mediated by the prolyl 4-hydroxylase-2 (PHD2) enzyme that regulates the stability of hypoxia-inducible factors (HIFs) [59]. In control subjects, VSMCs showed uniform expression pattern of PHD2 in the tunica media of leptomeningeal arteries (Fig. 6a, c). However, there was a decrease in PHD2 expression in the tunica media of leptomeningeal arteries in the human TBI, indicating increased levels of hypoxia (Fig. 6b, d). Under hypoxic conditions, VSMCs are known to undergo phenotypic changes from a contractile to proliferative synthetic phenotype [28]. To test this, we performed double staining using proliferating cell nuclear antigen (PCNA) and PHD2 (Fig. 6c, d). No detectable PCNA staining were found in the leptomeningeal arteries in control subjects (Fig. 6c). However, there were PCNA cells co-expressing PHD2 in the tunica media of human TBI subjects (Fig. 6d, e).

**Fig. 7:**
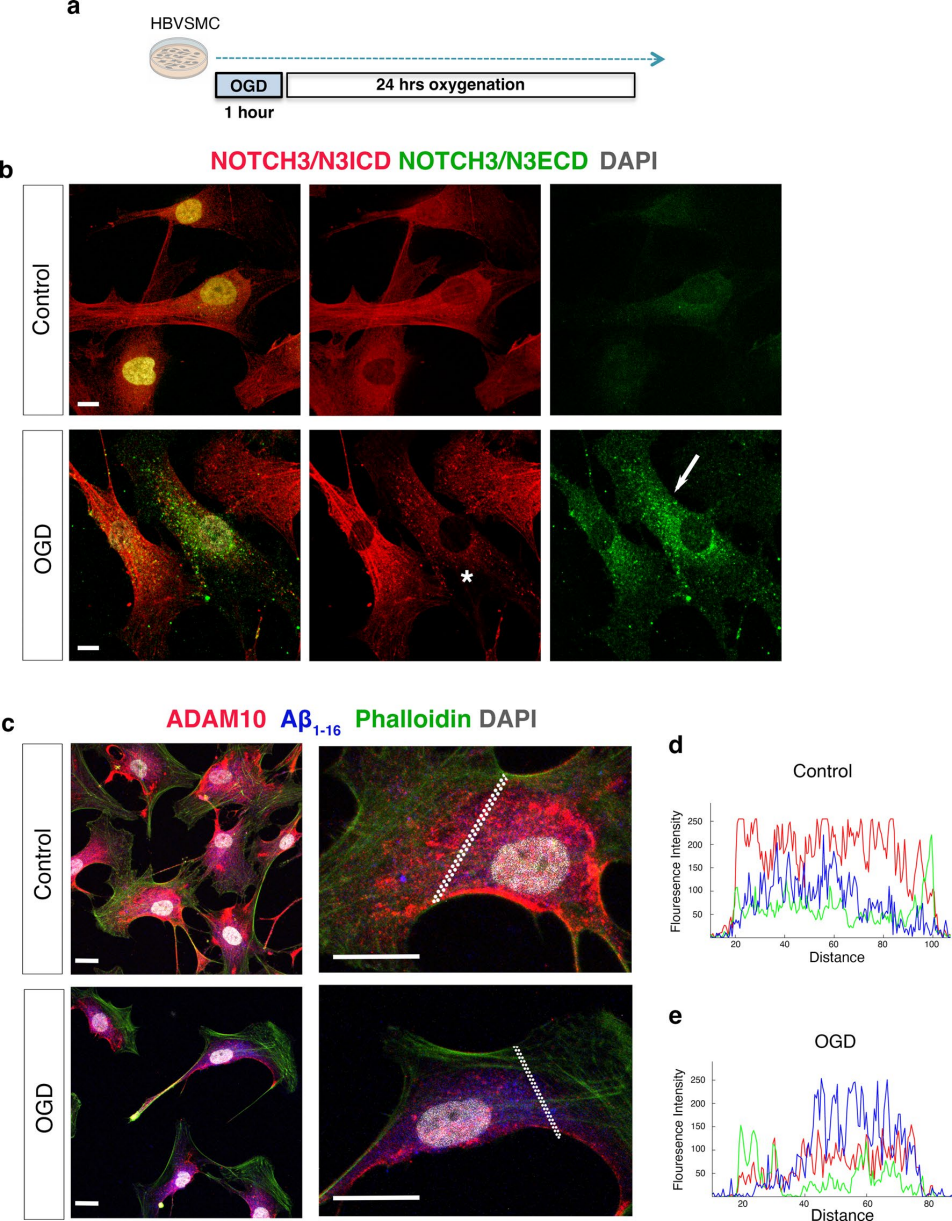
Hypoxia/oxidative stress in human VSMCs decreases NOTCH3/N3ICD and ADAM10 expression while increasing NOTCH3/ N3ECD aggregates and Aβ_1-16_. **a** The illustration shows the experimental design of oxygen and glucose deprivation /re-oxygenation (OGD/R) in vitro. **b** NOTCH3/N3ECD aggregates (green) on human VSMCs showing decreased NOTHC3/N3ICD levels (red) after exposure to OGD/R. **c** Confocal images of ADAM10 (red), Aβ_1–16_ levels (blue), and Phalloidin (green) staining in VSMCs exposed to OGD/R and in controls (**d–e**) The fluorescence intensity profiles of ADAM10 (red), Aβ_1–16_ levels (blue) and Phalloidin (green) in (**d**) control human VSMCs and (**e**) the ones exposed to OGD/R. Scale bars = 10 μm in (**b**, **c**)

**Fig. 8:**
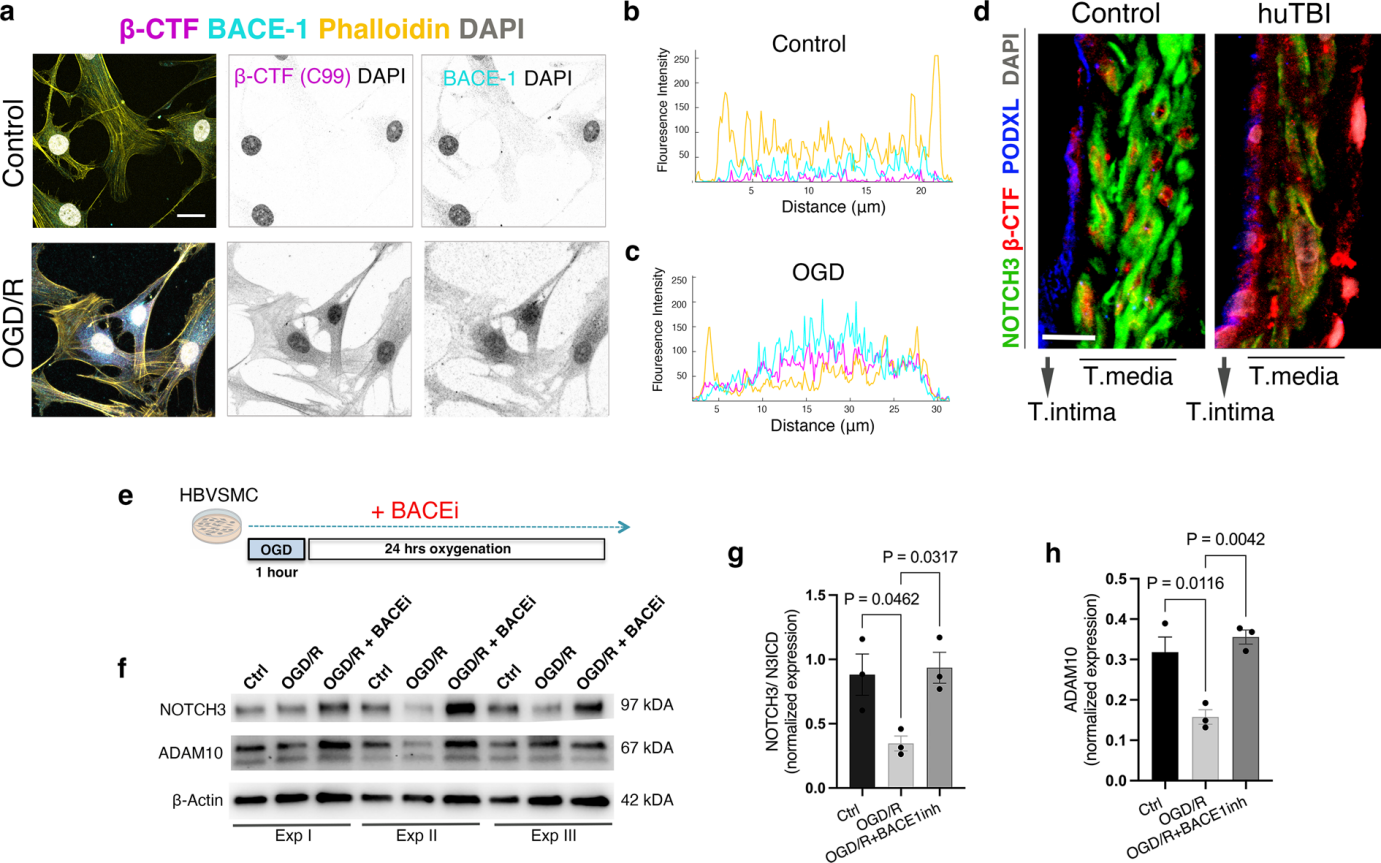
BACE1 inhibition restores NOTCH3 and ADAM 10 levels in human VSMCs exposed to oxygen and glucose deprivation /re-oxygenation (OGD/R). **a** Confocal images of immunofluorescent staining of βCTF (C99) (magenta), BACE1 (cyan), and in Phalloidin (yellow) in VSMCs in control and OGD/R, which were inverted into greyscale Scale bar = 10 μm. **b**–**c** The fluorescence intensity profiles of βCTF (C99) (magenta), BACE1 (cyan), and in Phalloidin (yellow) in VSMCs in (**b**) control and (**c**) OGD/R. **d** Confocal images of triple staining of NOTCH3 (green), βCTF (C99) (red), and podocalyxin (blue) in the leptomeningeal arteries of control and human TBI subjects. Scale bar = 10 μm (**e**) The illustration shows the experimental design of oxygen and glucose deprivation /re-oxygenation (OGD/R) in vitro and BACE1 inhibitor treatment of VSMCs. **f** Representative Western blots and (**g**–**h**) quantification of **g** NOTCH3 intracellular domain (NICD3) (97 kDA) and **h** ADAM10, active form (67 kDA) in VSMCs exposed to OGD/R and BACE1 inhibitor. (mean ± SEM; *n* = 3 (Ctrl), *n* = 3 (OGD/R), n = 3 (OGD/R + BACE1inh); one-way ANOVA followed by Tukey post hoc test, *P < 0.05)

We next aimed to dissect how TBI-induced hypoxia in the leptomeningeal arteries may affect the NOTCH3 expression and APP processing. To mimic the TBI-induced secondary injury in leptomeningeal arteries, we exposed human VSMCs to oxygen and glucose deprivation (OGD)/re-oxygenation (OGD/R) [58] (Fig. 7a). We used two antibodies that recognize both full length of NOTCH3 and the intracellular domain (N3ICD), and N3ECD, a truncated form of full length of NOTCH3 in the human VSMCs. We observed the presence of aggregates of NOTCH3 in the VSMCs with low expression of full length NOTCH3 after exposure to OGD/R (Fig. 7b). The fluorescence intensity of ADAM10 was decreased when human VSMCs were exposed to OGD/R compared to control conditions (Fig. 7c–e). On the other hand, there was an increase in the fluorescence intensity of Aβ1-16 (Fig. 7c–e), BACE1 as well as βCTF fragment of APP (C99) in VSMCs (Fig. 8a–c). Consistently, βCTF (C99) levels in NOTCH3 expressing VSMCs in the tunica media of arteries was higher in human TBI samples compared with control subjects (Fig. 8d).

Given that the reduction in ADAM10 expression in the VSMCs was more pronounced compared to BACE1, we hypothesized that higher BACE1 in the human VSMCs may affect the ADAM10 activity and NOTCH3 proteolysis under hypoxic/ischemic conditions. To test this hypothesis, we applied a highly selective BACE1 inhibitor, LY2886721, to human VSMCs during OGD/R exposure (Fig. 8e). Western blot analyses showed that the BACE1 inhibitor normalized the levels of NOTCH3 intracellular domain (NICD3) (*p* = 0.0317) at 97kDA (Fig. 8f, g) and increased the mature form of ADAM10 at 67kDA (*p* = 0.0042) in human VSMCs exposed to OGD/R) (Fig. 8f, h).

### NOTCH3 signalling and ADAM10 levels are reduced following diffuse TBI in mice

**Fig. 9:**
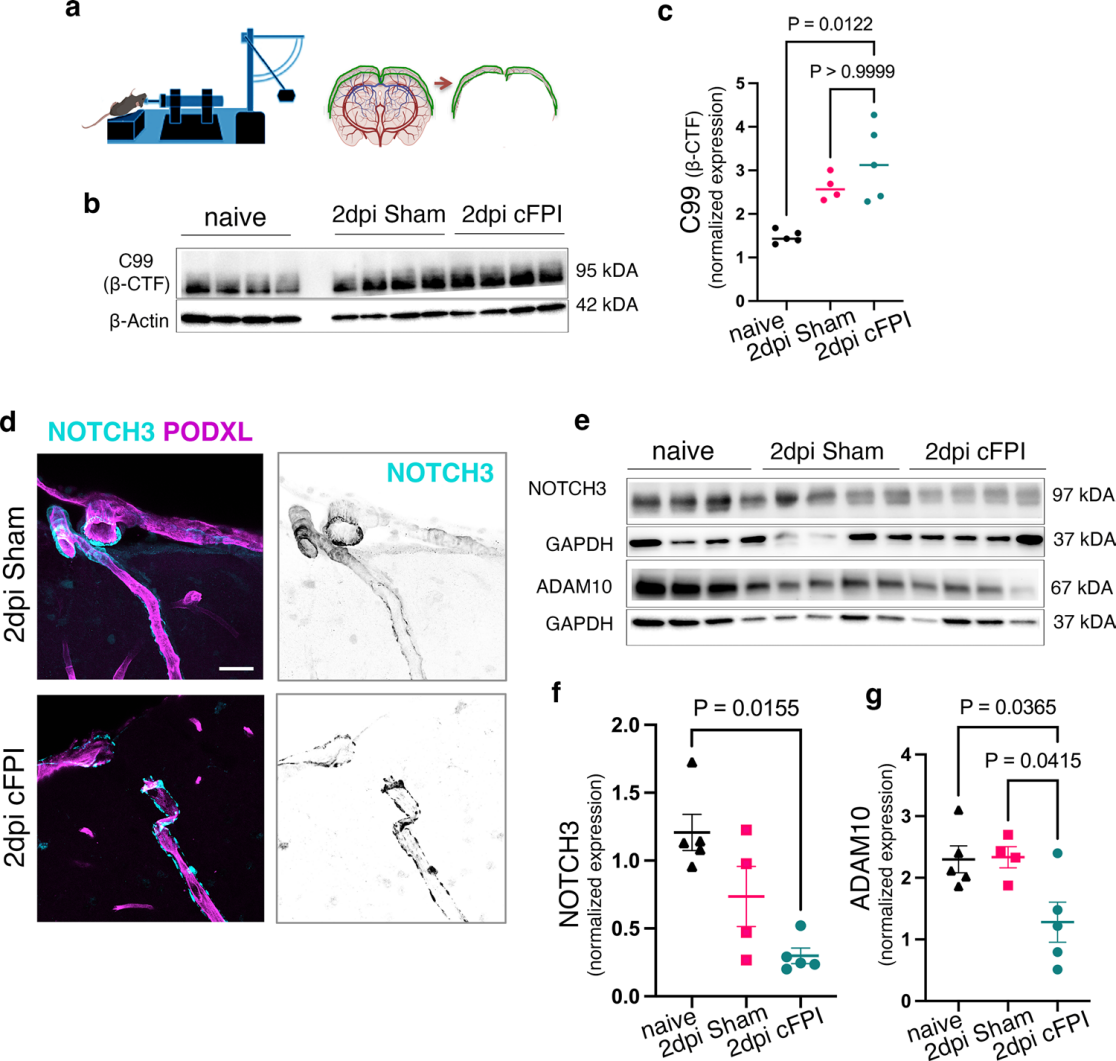
Experimental diffuse TBI causes a decrease in NOTCH3 signalling in leptomeningeal arteries accompanied by increased C99 levels and decreased ADAM10 activity. The illustration shows experimental design and the use of the central fluid percussion mouse model of TBI (cFPI; left) and isolation of sham or injured cortical consisting of leptomeningeal and penetrating leptomeningeal arteries. **b** Representative Western blots and (**c**) quantification of C99 (βCTF) band intensity in naïve, sham, and cFPI mice at 2 dpi (mean ± SEM; *n* = 5 (naïve), *n* = 4 (sham), *n* = 5 (cFPI); Kruskal–Wallis one-way ANOVA with Dunn’s post-test **P < 0.01). **d** Confocal images showing decreased NOTCH3 (magenta), expression on penetrating leptomeningeal arteries in the somatosensory cortex of sham-injured and cFPI-injured mice), NOTCH3 (magenta), and podocalyxin (cyan). Scale bar = 20 μm **e** Representative Western blots and **f**–**g** quantification of NOTCH3 intracellular domain (NICD3) (97 kDA) (**f**) and ADAM10, active form (67 kDA) (**g**) band intensity (mean ± SEM; *n* = 5 (naïve), *n* = 4 (sham), *n* = 5 (cFPI); ordinary one-way ANOVA followed by Tukey post hoc test, *P < 0.05)

Finally, we used the central (midline) fluid percussion (cFPI) model, which is a clinically relevant experimental model of diffuse TBI that subjects both axons and blood vessels to mechanical shearing forces and oxidative stress [18, 20, 51, 52]. In this mouse TBI model, we focused on an early, 2-day post-injury (dpi), time point to dissect the effect of cFPI on APP and Notch3 proteolytic processing. Protein lysates extracted from the upper surface of the cortex, which consist of both pial and descending pial arteries, were analysed by Western blotting (Fig. 9a). Consistent with human data, we found a significant increase in the levels of βCTF (C99) in cFPI mice, compared with naïve (*p* = 0.0122), but not compared with sham-injured mice (p > 0.999) (Fig. 9b, c). The leptomeningeal arteries, including surface and penetrating ones near the impact site, was properly visualized shortly after sham and cFPI (Fig. 9d). Notch3 immunoreactivity was found as deposits along the pial and penetrating arteries of cFPI-injured mice, whereas NOTCH3 staining showed a fibrous pattern wrapping around the arteries in the sham-injured and naïve mice (Fig. 9d, Fig.S2). Central fluid percussion resulted in an approximately fourfold decrease (*p* = 0.015) in the levels of NOTCH3-derived peptide containing the intracellular domain (N3ICD) at 97kDA, compared with naïve mice (Fig. 9e, f). Similarly, densitometry for ADAM10 (67 kDA) showed a significant decrease at 2dpi in lysates derived from cFPI animals compared with sham-injured (*p* = 0.041) and control mice (*p* = 0.036), indicating that these findings align with human TBI (Fig. 9e, g).

## Discussion

Traumatic brain injury (TBI) induces a series of pathological events that may initiate short- and long-term neurodegeneration because of cerebrovascular dysfunction and accumulation of pathological protein aggregates [12, 42]. The main findings of our present study were that TBI trigger early changes in VSMCs of leptomeningeal arteries, including NOTCH3 reduction and formation of Aβ peptides.

At time of TBI, the impact can cause vascular injury in leptomeningeal arteries and their collaterals in the subarachnoid space, or in capillaries of the brain parenchyma [38], leading to impaired cerebrovascular reactivity and reduced blood flow [4, 22, 30, 57]. These changes can have significant implications for cerebrovascular dynamics and blood flow e.g. in response to altered cerebrovascular reactivity to oxygen and glucose demands [27]. One possible hypothesis is that TBI may lead to changes in the structure of VSMCs on the walls of leptomeningeal arteries, as well as alterations in the composition and organization of the tunica media. The VSMCs in the tunica media are the major contractile components of the leptomeningeal arteries and play an important role in maintaining homeostasis of the cerebrovasculature [3]. Here, we showed that both human and experimental TBI resulted in a rapid decrease in NOTCH3 in the tunica media, a key signalling protein that regulates VSMC phenotype [15, 46, 47]. The decreased levels of NOTCH3 were closely associated with disorganization and detachment of VSMCs in the tunica media indicating that TBI may accelerate vascular degeneration similar to what occurs in the aging process [16, 34, 69].

The importance of NOTCH3 signalling in VSMCs haemostasis and function has been reported in other cerebrovascular diseases such as stroke, cerebral autosomal dominant arteriopathy with subcortical infarcts and leukoencephalopathy (CADASIL), and Alzheimer’s disease (AD), all characterized by amyloid beta (Aβ) accumulation in arteries and clinically with cognitive dysfunction [34, 69]. The accumulation of Aβ peptides in leptomeningeal and cortical arteries can lead to dilation of perivascular spaces and impaired drainage from the white matter, as reported in AD [55]. The current study revealed that Aβ peptides were rapidly formed in the arteries of human TBI subjects, preferentially around VSMCs in the tunica media [37]. Our findings align with previous studies suggesting that alterations in NOTCH3 cleavage may trigger the deposition of amyloid peptides in VSMCs, as observed in CADASIL and cerebral amyloid angiopathy [23, 33]. Furthermore, the reduction of NOTCH3 in VSMCs suggests that amyloid peptides in leptomeningeal arteries may result from NOTCH3-dependent VSMCs dysfunction leading to impaired periarterial drainage [3, 37]. Although the deposition of Aβ peptides was restricted to the tunica media in most leptomeningeal arteries, our results argue that the accumulation of Aβ protein can differ based on the size of arteries. This heterogeneous deposition of Aβ accompanied by reduced NOTCH3 can be due to other unknown factors affecting proteolytic cleavage of both NOTCH3 and APP. Nonetheless, this is the first study showing a close association between NOTCH3-related VSMC degeneration and Aβ deposition in the leptomeningeal arteries, particularly in young TBI patients [48]. This is an intriguing finding since it indicates that TBI-induced age-related degeneration processes may be initiated much earlier than previously believed.

NOTCH3 receptor is activated via ligand binding that trigger sequential cleavage in a manner comparable to that of APP processing [25]. Both ADAM10 and BACE1 are linked to vascular dysfunction through several mechanisms including angiogenesis. We previously reported that BACE1 activity was correlated with reduced NOTCH3 signalling in the vasculature when APP is overexpressed [16]. Moreover, TBI may induce hypoxia/oxidative stress, which results from mainly decreased CBF and oxygen supply to the brain [39, 40, 62]. Our finding shows that TBI-induced hypoxia/oxidative stress can further affect the proteolytic processing of NOTCH3 and amyloid beta processing. ADAM10 is particularly enriched at the cell surface and competes with BACE1 for APP processing. BACE1 is a beta-secretase that cleaves APP at the N-terminal end of the amyloid beta [70]. Under physiological conditions in the VSMCs, APP is cleaved through non-amyloidogenic pathway [13]. Upon TBI, higher levels of BACE1 may compete with ADAM10 to cleave APP to generate APP beta fragments and a membrane bound C99 fragment, activating amyloidogenic pathway. We showed that BACE1 expression was higher than ADAM10 expression in VSMCs, despite both being reduced in TBI subjects when compared to controls. This discrepancy may be attributed to TBI-induced hypoxia, which likely exacerbates the reduction of ADAM10 specifically in the VSMCs of leptomeningeal arteries [6]. TBI-induced hypoxia in the leptomeningeal arteries is a plausible upstream factor that contributes to changes in the expression of NOTCH3 as well as ADAM10 and BACE1, resulting in increased accumulation of Aβ peptides. This is consistent with our previous studies that human TBI specifically favour amyloidogenic processing, leading to the accumulation of soluble Aβ peptides, mainly N‐terminally intact and truncated Aβ_1−40/42_ [2, 43, 44]_._ However, smaller peptides such as Aβ_1-16_ as shown in this study could result from an alternative pathway during amyloidogenic processing, which involves the concerted action of ADAM10 and BACE1 [54]. Importantly, the increased production of βCTF(C99) in the human TBI samples, a diffuse experimental model of TBI and VSMCs in vitro, indicates activation of the amyloidogenic pathway. On the other hand, TBI-induced imbalance between ADAM10 and BACE1 as well as increased levels of Aβ fragments may inhibit the efficacy of γ-secretase and, in return, it can cause a decrease in NOTCH3 signalling [9, 16, 26]. Overall, our findings show that TBI-induced reduction in NOTCH3, and altered ADAM10 and BACE1 enzymatic activity in the presence of hypoxia, creates a detrimental feedback loop. As a result, the accumulation of Aβ peptides can further accelerate vascular dysfunction. However, TBI-induced vascular injury is a complex process that involves multiple mechanisms and pathways. Although microvessel alterations were not investigated in this study, we cannot exclude the possibility of an imbalance between ADAM and BACE1 which may lead to a decreased expression of endothelial nitric oxide synthase (eNOS), shifting the proteolytic cleavage of APP towards the amyloidogenic pathway [36]. On the other hand, ageing results in increased vascular expression of APP associated with the elevation of circulating soluble APP-alpha (sAPPα) [7, 17, 36]. Given that sAPPα has protective vascular properties, its potential for attenuating vascular injury related to amyloid-beta accumulation should be explored in the context of long-term TBI.

TBI-induced damage to the leptomeningeal and penetrating arteries can influence subcortical areas through various interconnected pathologies [68]. Therefore, the cFPI model we used in this study is clinically relevant as it enables the examination of pathological changes in brain regions not directly targeted by the physical impact of the delivered fluid pressure pulse [41, 52]. This also aligns with previous studies that reported the fluid percussion models in rodents are effective tools to investigate acute post-traumatic hypoperfusion and its association with disrupted vascular integrity and decreased capillary density [29, 49, 53].

Finally, human TBI manifests in different severities, from mild to severe, including blast-induced brain injury, concussion, focal TBI, and diffuse injury. Each subtype of TBI shows distinct yet interconnected effects on brain vasculature [19, 20, 65]. Importantly, the vascular injury resulting from all different subtypes of TBI can profoundly impact CBF, potentially influencing long-term neurologic outcomes. In sports-related concussion, early and late changes in CBF have been observed [35, 62]. High-pressure blast waves can lead to acute vascular injury in both humans and in experimental animals, and leptomeningeal haemorrhage has been observed in severe TBI patients [19, 21]. On the other hand, recent clinical studies using non-invasive imaging techniques, such as arterial spin labelling (ASL) magnetic resonance imaging (MRI), have demonstrated CBF changes in both the acute and chronic phases of TBI [10, 38, 56, 67]. Specifically, acute TBI patients show regions of either hypo- or hyperperfused brain regions when compared to healthy controls [67]. However, the development of acute TBI treatments still encounters obstacles due to a lack of reliable molecular-based diagnostics. Our study suggests that targeting NOTCH3 pathway together with APP processing could be a promising therapeutic strategy for preserving VSMC identity and maintaining cerebrovascular function during acute and chronic phases of TBI.

The present study has several limitations. In view of the emergent and critical nature of the included TBI patients, they did not undergo evaluation of CBF by e.g. ASL MRI or Xenon-CT [56, 65, 67]. However, in severe TBI, an early reduction in cerebral blood flow (CBF) is well-established and in severe TBI patients dying from their injuries, hypoxic injuries are widespread. Thus, while a direct correlation of CBF or impaired autoregulation to the immunohistochemical finding could thus not be performed here, an alteration of cerebrovascular function is likely. The TBI tissue samples used in this study were obtained from rather rare cases of neurosurgically resected TBI tissue from emergency surgery procedures. Thus, the tissue was obtained from the region of most severe injury and partially consisted of some haemorrhagic and necrotic tissue. On the other hand, the control subjects were derived from post-mortem tissue obtained from acute, non-neurological deaths. This discrepancy may affect the comparability of TBI samples. As a result, the samples may differ when analysing protein levels, such as amyloid protein, ADAM10, and BACE1. However, we addressed this challenge using an experimental model of diffuse TBI and by in vitro studies, which allowed us to further validate our results and strengthen our conclusions. By applying the OGD/R in vitro model of VSMCs, we aimed to mimic the ischemic conditions that may occur in the leptomeningeal arteries during TBI due to reduced cerebral blood flow. However, TBI involves mechanical forces not accounted for in OGD/R, which requires modifications for future experiments.

## Supplementary Information

Below is the link to the electronic supplementary material.

Supplementary file1 (DOCX 6669 KB)

## Data Availability

No datasets were generated or analysed during the current study.
