# Supplementary material for: Traumatic brain injury causes early aggregation of beta-amyloid peptides and NOTCH3 reduction in vascular smooth muscle cells of leptomeningeal arteries
Source: Acta Neuropathol. 2025 Jan 22;149(1):10. doi: 10.1007/s00401-025-02848-9 (PMC11754316; doi:10.1007/s00401-025-02848-9)

**Supplementary Information.**

**Supplementary Table 1.**  Patient characteristics traumatic brain injury

| **Patient #** | **Age** | **Gender** | **Cause of injury** | **Other injuries** | **Time post-injury (h)** | **Region of surgery** | **Surgery** | **mGCS**  **pre-op** | **Histology** | **GOSE** |
| --- | --- | --- | --- | --- | --- | --- | --- | --- | --- | --- |
| **#3**  **#4** | 42  65 | M  M | MVA  Fall | None  None | 4  193 | RF  LT | Ccx  CCx | 5  1 | NA  AS | 8  NA |
| **#6** | 74 | M | Fall | None | 4 | RT | Ccx | 5 | Aß | 4 |
| **#7** | 58 | M | Fall | Efx | 9 | LT | Ccx | 5 | No ND | 7 |
| **#8** | 49 | M | Fall | None | 84 | RT | Ccx* | 5 | No ND | 1 |
| **#9** | 19 | F | Fall | None | 16 | RFT | Ccx + DC^^ | 3 | No ND | 3 |
| **#10** | 65 | M | Fall | Ffx, Thi | 180 | LT | Ccx | 3 | Aß | 3 |
| **#11** | 25 | M | SPR | None | 24 | LFP | Ccx + DC** | 2 | AS | 3 |
| **#14** | 67 | M | SBO | None | 4 | LT | Ccx | 5 | Aß | 1 |
| **#16** | 52 | M | Fall | None | 42 | RT | Ccx*** | 4 | No ND | 4 |
| **#18** | 54 | M | ASL | None | 312 | LF | Ccx**** | 5 | AS, HpT | 7 |
| **#19** | 49 | F | HBO | None | 57 | RF | Ccx*** | 2 | No ND | NA |
| **#20** | 62 | F | MVA | None | 192 | RF | Ccx*** | 4 | AS, HpT | 8 |
| **#21** | 24 | M | Fall | None | 106 | RF | Ccx | 5 | AS, HpT | 6 |
| **#22** | 49 | F | Fall | None | 32 | LFT | Ccx + DC^^ | 5 | No ND | 1 |

*coagulopathy

^^ DC prior to CCx, performed at re-surgery

** Initial surgery for aSDH and DC in primary hospital, CCX + revised DC secondary surgery

*** Initial surgery for aSDH, Ccx at re-surgery

**** Combined surgery for aSDH and CCx

DC=Decompressive craniectomy; M= male; F= female; L= left, R= right; T= temporal; F= frontal, P= parietal, Ccx= removal of cortical contusion; mGCS; motor component of the Glasgow Coma Scale; SPR: sports-related; MVA= motor-vehicle accident; SBO= struck by object; ASL= Assault; Thi= thoracic injury; Efx= extremity fracture; Ffx= facial fracture, GOS= Glasgow Outcome Scale, Aß= beta-amyloid plaques, AS = axonal swelling; HpT= Hyperphosphorylated Tau; NA= not available, ND = neurodegeneration, GOSE=Glasgow outcome scale extended

**Supplementary Figure 1.**  An extended representative confocal image for Fig. 1. Triple fluorescence staining of NOTCH3(red), podocalyxin (blue) and DAPI (grey) in the leptomeningeal arteries of human TBI subjects stained with DAB Fig. 1d.


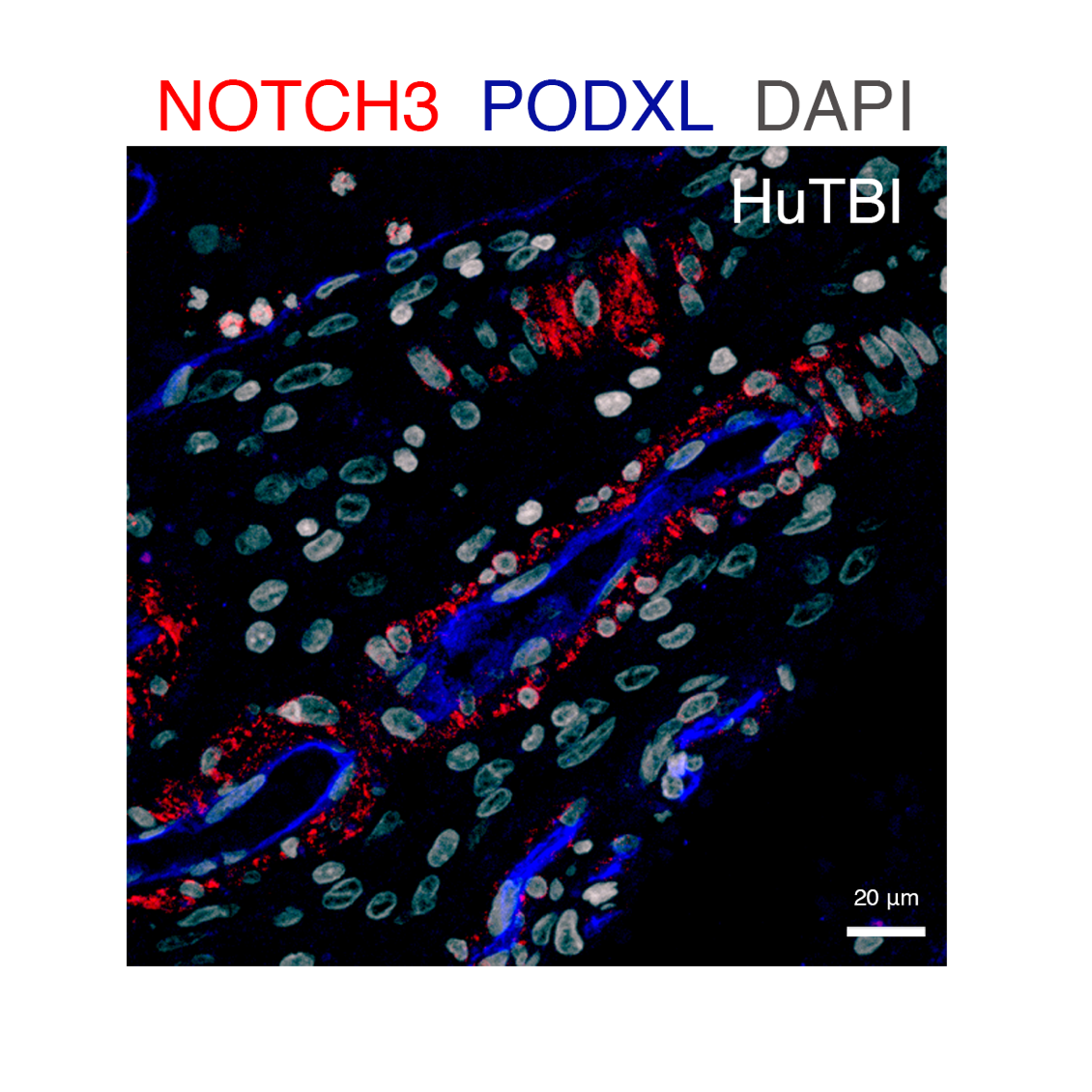


**Supplementary Figure 2.**  An extended representative confocal image for Fig. 8d. Triple fluorescence staining of NOTCH3(red), podocalyxin (blue) and α-SMA (green) in the pial arteries of naïve mice.


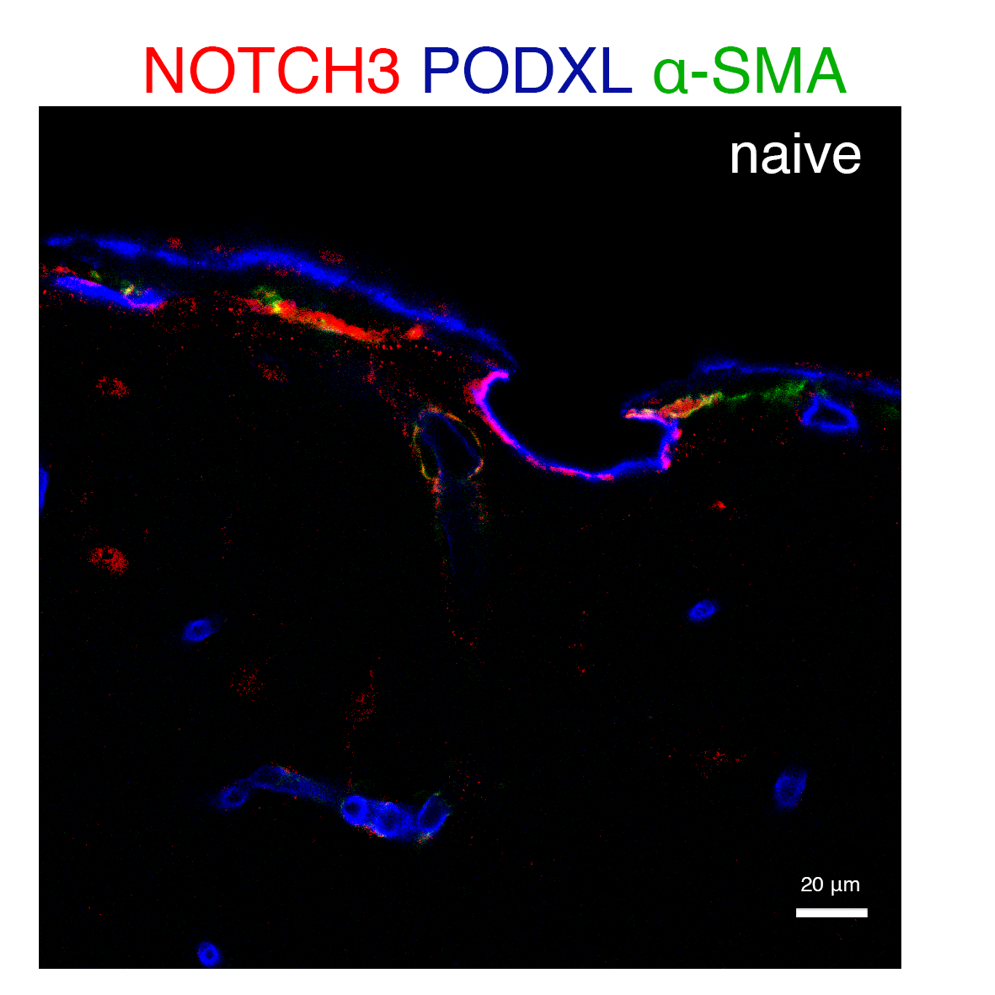

Supplement: Supplementary file 1 — Supplementary file1 (DOCX 6669 KB) [file 401_2025_2848_MOESM1_ESM.docx]
